# Intranasal vaccination with messenger RNA as a new approach in gene therapy: Use against tuberculosis

**DOI:** 10.1186/1472-6750-10-77

**Published:** 2010-10-20

**Authors:** Julio CC Lorenzi, Ana PF Trombone, Carolina D Rocha, Luciana P Almeida, Ricardo L Lousada, Thiago Malardo, Isabela C Fontoura, Renata AM Rossetti, Ana F Gembre, Aristóbolo M Silva, Celio L Silva, Arlete AM Coelho-Castelo

**Affiliations:** 1 Departamento de Bioquímica e Imunologia, Faculdade de Medicina de Ribeirão Preto, Universidade de São Paulo, Brazil; 2 Departamento de Morfologia, Instituto de Ciências Biológicas, Universidade Federal de Minas Gerais. Belo Horizonte, Minas Gerais, Brazil

## Abstract

**Background:**

mRNAs are highly versatile, non-toxic molecules that are easy to produce and store, which can allow transient protein expression in all cell types. The safety aspects of mRNA-based treatments in gene therapy make this molecule one of the most promising active components of therapeutic or prophylactic methods. The use of mRNA as strategy for the stimulation of the immune system has been used mainly in current strategies for the cancer treatment but until now no one tested this molecule as vaccine for infectious disease.

**Results:**

We produce messenger RNA of Hsp65 protein from *Mycobacterium leprae *and show that vaccination of mice with a single dose of 10 μg of naked mRNA-Hsp65 through intranasal route was able to induce protection against subsequent challenge with virulent strain of *Mycobacterium tuberculosis*. Moreover it was shown that this immunization was associated with specific production of IL-10 and TNF-alpha in spleen. In order to determine if antigen presenting cells (APCs) present in the lung are capable of capture the mRNA, labeled mRNA-Hsp65 was administered by intranasal route and lung APCs were analyzed by flow cytometry. These experiments showed that after 30 minutes until 8 hours the populations of CD11c^+^, CD11b^+ ^and CD19^+ ^cells were able to capture the mRNA. We also demonstrated *in vitro *that mRNA-Hsp65 leads nitric oxide (NO) production through Toll-like receptor 7 (TLR7).

**Conclusions:**

Taken together, our results showed a novel and efficient strategy to control experimental tuberculosis, besides opening novel perspectives for the use of mRNA in vaccines against infectious diseases and clarifying the mechanisms involved in the disease protection we noticed as well.

## Background

The use of mRNA to encode a protein as a vehicle in gene therapy was extensively used in cancer research [1]. This method relies on the in vitro transfection of mRNA into autologous dendritic cells (DCs) that are re-administered to the patient. It has shown good efficiency in terms of induction of T-cell responses in cancer patients, but presented several drawbacks including cost, limited number of cells/vaccine doses, and the intrinsic phenotypic variability of the in vitro generated DCs. Moreover, the ideal maturation state and delivery modalities of transfected DCs are still matter of debate. As an alternative, mRNA could be used in naked form. Direct injection of mRNA coding for immune dominants antigens could lead a strong immune response against the encoded antigen, as demonstrated in cancer models [2,3]. Recently, Weid [4] concluded the second phase 1/2 vaccination trial in metastatic melanoma patients. Using mRNA from six different immune dominant antigens from melanoma he showed that after vaccination patients developed important CD4^+ ^immune response. One important feature of naked mRNA is the ability of fast activation of the innate immune system by pathways such as Toll like receptors (TLRs) [5], RIG 1 and MDA5 [6]. Moreover, the action of the translated protein encoded by the naked mRNA can also take part in the induction of specific immune response.

Tuberculosis (TB) causes more than 2 million deaths per year all over the word [7]. Currently the only available tuberculosis vaccine is *Mycobacterium bovis *bacillus Calmette-Guerìn (BCG), but its effectiveness varies, particularly against the adult pulmonary form of the disease [8]. The increasing incidence of AIDS, long chemical treatments and the emergence of multidrug resistant strains are compelling evidences for the urgency of new therapeutic strategies against TB [9]. Since its first description in March, 2006 [10], resistance has become the most alarming issue in international tuberculosis control and might be responsible for compromising the progress observed in many countries over the last decade [11].

Many studies of subunit vaccines have been undertaken by using different antigens, such as Ag85 complex [12], 38 kDa protein [13], 19 kDa protein [14], RD1 region [15] and Ag85B-ESAT-6 complex [16]. It has been reported that 65 kDa heat shock protein (Hsp65) is one of the major immune reactive proteins during the *Mycobacterium tuberculosis *(MTB) infection. Additionally, the immunity induced by DNA vaccines encoding *M. leprae *Hsp65 (DNA-Hsp65) can reduce the bacterial loads in spleen and lung of the infected mice after intramuscular injection [17,18]. The immunity induced by Hsp65 reflects mainly in the production of Th1 cytokines mostly IFN-gamma [19]. Moreover, it was well demonstrated that DNA vaccines encoding MTB Hsp65, initially designed to prevent infection, also had a pronounced therapeutic effect, which could eliminate the bacteria in TB mice after antibacterial chemotherapy [20]. The antigens cited above have already been tested using plasmid DNA constructs. Once plasmid DNA is the major contributor in the TB vaccine list, some issues concerning security, like the possibility of DNA integration and autoimmunity, cannot be assured [21]. In this way, the development of a new strategy based on messenger RNA (mRNA) can be an attractive approach. We show here that one intranasal dose of 10 μg of mRNA coding for Hsp65 protein from *M. leprae *leads to an immune response that protects mice against *M. tuberculosis *infection.

## Results

### Expression profile of mRNA-Hsp65 produced in vitro

**Figure 1:**
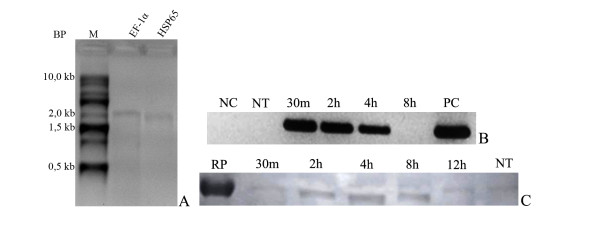
**(A) - Electrophoretic profile of *in vitro *synthesized messenger RNAs**. Lane M: 0.5-10 kb RNA Ladder (Invitrogen), lane 1: 1 μg of mRNA-Hsp65, 2: 1 μg of EF-1α mRNA. The electrophoresis was performed in 1.5% denaturing agarose gel stained with ethidium bromide. (BP-Base pair, M-Marker). (**B) **- Detection of the integrity of the mRNA-Hsp65 by RT-PCR. After transfection of HEK 293 cells with the mRNA-Hsp65 cells were maintained in culture for different periods of time, as showed in the figure, in order to analyze the presence of mRNA-Hsp65. Electrophoresis was run in 1.5% agarose gel and stained with ethidium bromide (NC-Negative PCR control, NT-Not transfected, PC-Positive PCR control). (**C) - **After contact mRNA-Hsp65 for different periods of time, the total cell lysate was subjected to polyacrylamide gel electrophoresis (12.5%) and the bands transferred to nitrocellulose membrane and incubated with anti-Hsp65 for 2 hours. The reaction was revealed with secondary antibody anti-mouse IgG in the presence of DAB. (RC-Recombinant protein Hsp65, NT-Not transfected)

The first step in the protocol of the mRNA vaccine was to evaluate if the mRNA production had been successful. As shown in figure 1(A), the purified mRNA-Hsp65 and EF-1α mRNA showed a fragment of 1,800-bp (lane 1) and 1,850-bp (lane 2), respectively, which matched the expected length. When the mRNA-Hsp65 and the control mRNA were treated with RNAse A, no fragment was observed (data not shown), confirming the homogeneity of the mRNA preparation. In order to verify the presence of possible sites that could inhibit the translation of mRNA-Hsp65, we also determined the RNA-Hsp65 predicted structure using Mfold web based software[22] (Additional file 1). These analyses showed that mRNA-Hsp65 did not have significant loops or arms that could interrupt the translation process.

To determine if mRNA-Hsp65 could be translated in mammalian cells, we first detected mRNA and protein in mRNA-Hsp65-transfected HEK293T cells. The Figure 1(B) RT-PCR and 1(C) western-blot confirms the mRNA stability and the Hsp65 protein expression in HEK293T cells at various time points after the mRNA uptake by these cells. After 30 minutes, 2 and 4 hours of the incubation we detected mRNA-Hsp65 by RT-PCR (Figure 1B). Presumably mRNA was consumed or degraded by the cell metabolism after 8 hours from the inoculation of the mRNA, as observed by the lack of intensity of gel fragment. The protein expression was observed after 30 minutes of inoculation with mRNA-Hsp65 and persisted until 12 hours later (Figure 1C, densitometry in Additional file 2). These results confirmed that mRNA-Hsp65 is stable inside mammalian cells and could be translated in Hsp65 protein.

### Lung antigen presenting cells (APCs) can uptake mRNA-Hsp65 in vivo after intranasal immunization

**Figure 2:**
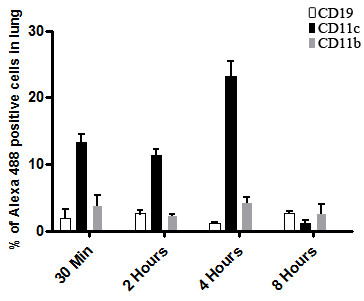
**Assessment of the amount of professional antigen-presenting cells capable of capturing mRNA-Hsp65 labeled with Alexa488**. Five BALB/c mice per group were immunized by intranasal route with one dose of 10 μg of Alexa488 labeled mRNA-Hsp65. The control group received Ringer's solution. The kinetics of the capture was made in different times (indicated in the legend) and the cellular phenotype detected by flow cytometry. Data represent the mean positive cell counts ± SD of five mice per group of one of three independent experiments

Once mRNA was captured and translated *in vitro*, we evaluated if mRNA-Hsp65 could be uptaken by mammalian cells *in vivo*. To test this hypothesis, mice were intranasally immunized with tagged mRNA-Hsp65. After different time points lung cells were analyzed by flow cytometry. From 30 minutes until 4 hours post-immunization we observed that dendritic cells were the majority of cells that had uptaken the mRNA-Hsp65 when compared with macrophages and B cells. Four hours after the immunization around 25% of dendritic cells were positive for the tagged mRNA (Figure 2). Besides the uptake by classical APCs we do not detected non APCs cells marked for tagged mRNA-Hsp65 (Additional file 3). Taken together these data confirmed that intranasal route could be used for mRNA vaccine strategy, since these molecules were able to reach lung APCs.

### Vaccination with mRNA-Hsp65 induces protection against tuberculosis

**Figure 3:**
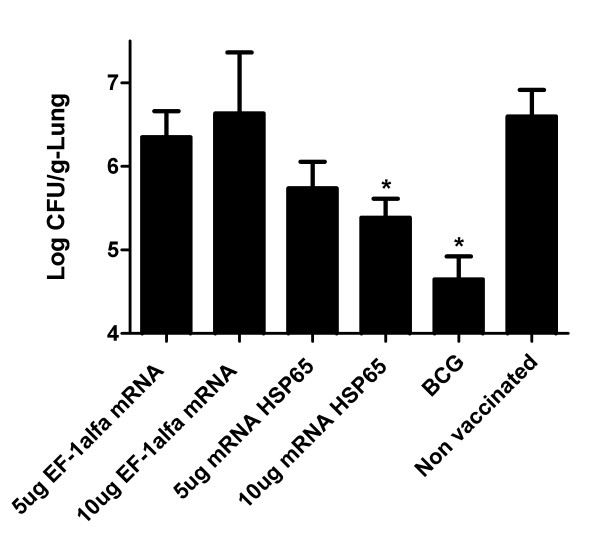
**Intranasal vaccination with mRNA-Hsp65 protects mice against tuberculosis**. Five BALB/c mice per group were immunized by intranasal route with a dose of different formulations containing mRNA-Hsp65 or EF-1α mRNA. BCG group received subcutaneously 1 dose of BCG Moreau. Non vaccinated group received only the Ringer's solution. Thirty days after the last immunization, animals were challenged with 10^5 ^bacilli of the virulent H37Rv strain of *M. tuberculosis *by intranasal route. Thirty days after challenge, animals were euthanized and their lungs extracted for the evaluation of the bacterial load. Data represent the mean log10 CFU counts ± SD of five mice per group of one of three independent experiments. *p < 0.05 were considered significant when compared to non vaccinated group.

**Figure 4:**
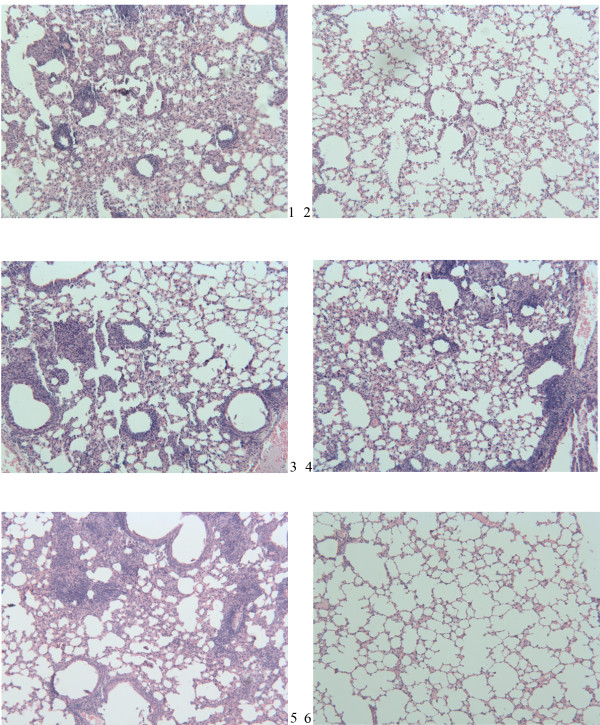
**Histological analysis of lungs of BALB/c mice vaccinated and challenged with virulent strain of *Mycobacterium tuberculosis***. Five BALB/c mice per group were immunized by intranasal route with a dose of different formulations containing mRNA-Hsp65 or EF-1α mRNA. The BCG group received 1 dose of BCG Moreau subcutaneously. Thirty days after the last immunization animals were challenged with 10^5 ^bacilli of the virulent strain of *M. tuberculosis *by H37RV intranasal route. Thirty days after challenge, animals were sacrificed and their lungs extracted for the recovery histology. 1-Non vaccinated group, 2-BCG group, 3-EF-1α mRNA 5 ug group, 4-EF-1α mRNA 10 μg group, 5-mRNA-Hsp65 5 ug group, 6-mRNA-Hsp65 10 μg group. Staining was performed with hematoxylin and eosin (HE), increased original image of 100×.

In order to verify if mRNA-Hsp65 vaccination strategy works *in vivo*, we tested this strategy in tuberculosis experimental model. Thus, we challenged mice by intranasal route with virulent *M. tuberculosis *H37Rv 30 days after immunization with the 10 or 5 μg of mRNA-Hsp65 or control EF-1α mRNA or BCG. Four weeks after the challenge with *M. tuberculosis*, bacterial loads in the lungs were quantified by CFU protocol. Only mice that were immunized with 10 μg of mRNA-Hsp65, showed a significant decrease of bacterial count when compared with the mRNA control or the RL control group (p < 0.05) (Figure 3). The BCG group was used as a positive protection control. Histological lung sections from infected mice were analyzed to determine the development and the presence of the inflammatory infiltrates. As shown in figure 4, mice treated with 10 μg mRNA-Hsp65 vaccine (panel 6) or BCG (panel 2) showed almost intact alveolar tissue. On the other hand, there was considerable infiltration of mononuclear cells and extensive parenchyma destruction evidenced by large and poorly demarcated granuloma in the lungs from the control mice immunized only with 5 or 10 μg of EF-1α mRNA (panel 3 and 4).

### Immunization with mRNA-Hsp65 induces production of IL-10 and TNF-alpha cytokines

**Figure 5:**
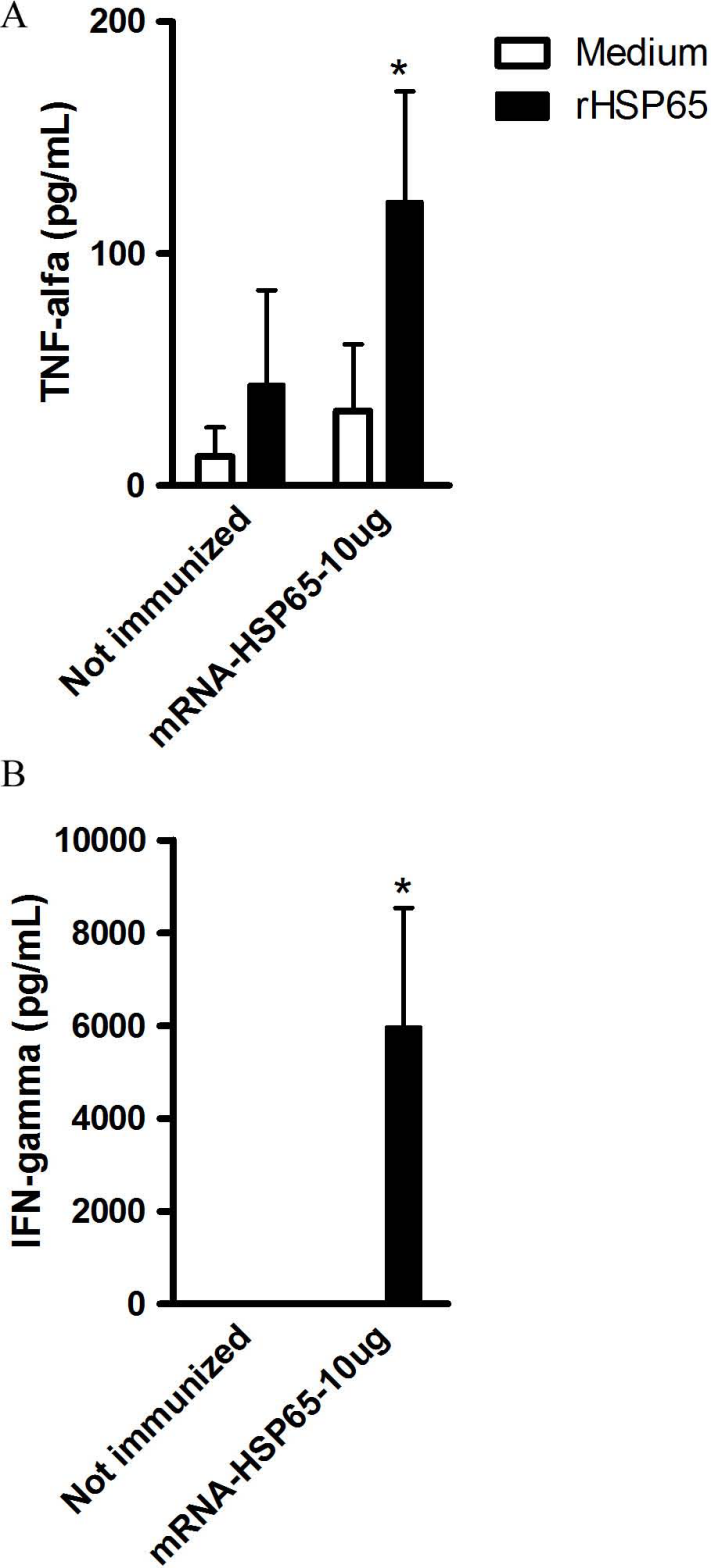
**Immunostimulatory activity of mRNA-Hsp65**. Five BALB/c mice per group mice were immunized with one dose of mRNA-Hsp65 (10 μg per mouse in 100 μL volume). Not immunized group received only the Ringer's solution. Two weeks after the immunization splenic cells are isolated and stimulated with 20 μg/ml of Hsp65 protein, 48 hours after stimulation the production of (A) IFN-gamma and (B) TNF-alpha by were determined by ELISA. *p < 0.05 were considered significant when compared to not immunized group.

Cytokines play an important role in the immune response to *M. tuberculosis*, since it was demonstrate that vaccination with mRNA-Hsp65 induces protection against tuberculosis we explore if the immunization with mRNA-Hsp65 generated specific Th1 cytokine production. We observed that immunization with 10 μg of naked mRNA-Hsp65 induced a specific and significant increase of IFN-gamma (figure 5A) and TNF-alpha (figure 5B) compared to RL control group. Concanavalin A (ConA) stimulation was used as control. Cytokine production obtained with ConA was shown in Additional file 4.

### mRNA-Hsp65 stimulation lead NO production via Toll like Receptor 7

**Figure 6:**
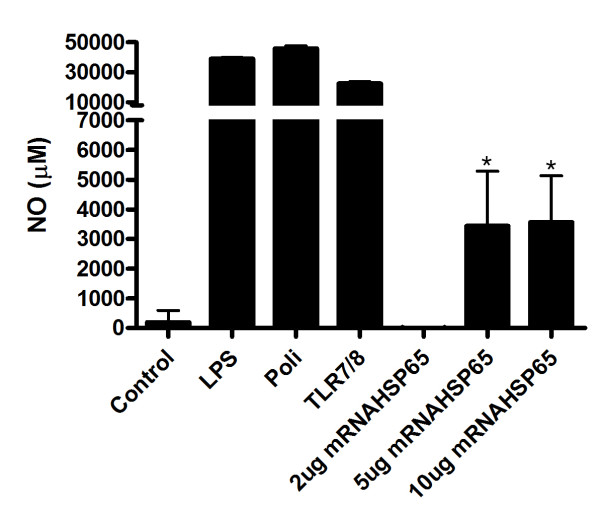
**TLR7 activation by hsp65 mRNA**. HEK293T cells (3 × 10^5 ^cells/well) were co-transfected in triplicate with the TLR expression plasmids. The next day, cells were treated with hsp65 mRNA or following TLRs agonists: poly-IC 100 μg/ml (TLR3), LPS 1 μg/ml (O55:B55) (TLR4) and R-848 1 μg/ml (TLR7 and 8) for 7 hours. The amount of NO release was measured form culture supernatant using the method of Griess. Data are presented as the means ± SD of triplicate cultures. *p < 0.05 were considered significant when compared to empty vector.

Once it was shown that mRNA-Hsp65 could induce protection against *M. tuberculosis *challenged *in vivo*, we explored if this effect could be started by TLR7 activation. Thus, HEK293T cells were transfected with TLR 7 and exposed to mRNA-Hsp65 *in vitro*. Measuring NO release from these cells, we showed that mRNA-Hsp65 stimulates the production of this molecule significantly via TLR 7 receptor (Figure 6).

## Discussion

We showed here that one dose of 10 μg of naked mRNA encoding *M. leprae *Hsp65 was enough to protect mice against *M. tuberculosis *infection. We also showed that this naked mRNA stimulated Th1specific cytokine production and was efficiently captured by different lung APCs after intranasal immunization, mainly by dendritic cells and. Moreover we detected activation of TLR7 pathway via mRNA-Hsp65.

The use of naked mRNA as a vehicle for gene therapy has increasing in the last few years. The majority of the studies used mRNA transfected in dendritic cells and were focused on the stimulation of CD8^+ ^cytotoxic T lymphocyte response, as well as on the induction of CD4^+ ^T helper cell response, in order to obtain optimal and sustained immune responses capable of eliminating tumor cells [23]. Although the use of naked mRNA was more restricted to cancer research until now, in this report we showed that it is possible to use naked mRNA intranasally against a pulmonary disease. This mRNA can pass through all nasal and tracheal barriers and reach the lungs, where they can be captured by different APCs. In our approach we used an mRNA from *M. leprae *Hsp65, which has already been extensively evaluated as an experimental tuberculosis vaccine, an mRNA capable of being translated into transfected mammalian cells. In HEK 293T cells transfected with mRNA-Hsp65 the protein expression was observed since the early 2 hours of the delivery of the mRNA, until 12 hours after transfection. These results showed that any possible differences in protein expression of prokaryotic system like codon bias [24] did not suppress the expression of *M. leprae *protein in mammalian system. Also, mRNA-Hsp65 was stable and ready for expression in mammalian system, suggesting that the formulated vaccine mRNA can be kept inside HEK 293T cells until its translation. Besides, we used bioinformatics tools to show that produced mRNA-Hsp65 had a minimum structure that would not interfere in the translation process, since this parameter is very important to initiate the protein production [25]. We also confirmed that once the mRNA was inside the cell, the translation process could be done, with the production of Hsp65 30 minutes after the transfection process (Figure 1C). This result could explain the functionality of the vaccination, once we observed protein production and protection against *M. tuberculosis *infection in mice. In addition, the induction of specific immune response against the mRNA encoding antigen, can also be useful in gene therapy because this kind of mRNA have some structural characteristics that confer the ability to activate APCs [26].

After the translation process, the produced protein can enter in the class I pathway of immunologic presentation process for activation of CD8^+ ^T cells or participate of cross-priming process for CD4^+ ^T cells [27]. The mRNA performs this activation process and in the maximum of 8, 12 hours disappears of the system. This interesting characteristic differs from other gene therapy vehicles such as plasmid DNA vaccines that can be rescued in the *in vivo *system after 6 months of immunization [28]. In addition to mRNA stability, we showed the uptake of this mRNA by lung APCs, mainly dendritic cells, after the intranasal immunization, suggesting that transfected dendritic cells may induce a protective immune response. The activation state of these cells was critical in the tuberculosis infection since macrophages and dendritic cells can stop the infection in the early stages, if stimulated. Moreover after the immunization this APCs can stimulate specific T cells, creating memory T cells [29].

The CFU counting and histopathology analysis showed that 10 μg of mRNA-Hsp65 given by the intranasal route can reduce the bacterial load and inflammation in mice lungs. When we compared the CFU results from mice immunized with the control mRNA containing the mRNA-Hsp65 group it is possible to reaffirm that Hsp65 is a vaccine antigen, since the control mRNA group neither decreased the bacterial load nor improved the lung inflammation as compared with RL group. We speculated that only innate cellular activation provided by the control mRNA was not able to protect mice against tuberculosis.

The protection that we obtained in our experiments was very motivating since we used only one dose of 10 μg of mRNA-Hsp65 by the intranasal route, contrasting the use of three doses of 100 μg plasmid DNA by intramuscular route in the original research [18] passing by prime-boost [30] and recently with entrapment of plasmid DNA in cationic vesicles reduce to an single dose of 25 ug of plasmid by the intranasal route [31]. Besides, we used a needle free immunization, intranasal, which has a great advantage in the development of a TB vaccine since the nasal associated lymphoid tissue participate in the initiation and modulation of immune response against pulmonary infections [32].

Moreover we showed that immunization with mRNA-Hsp65 could induce specifically production of Th1 cytokines IFN-gamma and TNF-alpha. IFN-gamma are essential for the development of protective immunity against TB [33,34] and is probably the most important factor that activates macrophages antimycobacterial action, at least in TB response [19,35]. The importance of TNF-alpha in TB improved since the discovery of reactivation of dormant TB in patients using TNF-neutralizing drugs [36,37]. From these studies the role of TNF in the formation of granuloma was demonstrated [37]. TNF-alpha blockage impair the antimicrobial activity of memory CD8^+ ^T cells by reducing the production of perforin and granulysin the major components of CD8^+ ^T antimicrobial response to intracellular pathogens [38]. Thus the mRNA approach could induces a TH1 pattern like DNA vaccines.

Furthermore the presence of important cytokines after mRNA-Hsp65 immunization, driven by the expression of Hsp65 protein, we asked if this mRNA molecule could induce intracellular activation via toll like receptor 7. Through *in vitro *experiments we showed that mRNA-Hsp65 binds TLR7 leading the production of NO, an antimicrobrial molecule that is induced by TLR [39]. The activation via TLR7 culminates in NF-KB expression leading production of inflammatory cytokines. This process could occur simultaneously with the Hsp65 protein production, which may be considered a potent co-stimulatory event that enhances the immune activation of Hsp65 protein.

In the last two decades, tuberculosis incidence changed from a disease exclusive of poor countries into a worldwide illness. This change becomes visible with the presence of multi-resistance and extreme resistant strains of *M. tuberculosis *all over the word [40]. Thus, the search for a new vaccine against TB became a very important issue. But until now the only available vaccine is BCG that has been extensively used, but is not always effective. Our group has worked on the development of a tuberculosis vaccine using different approaches that could deliver a well know and effective antigen to the immune system [41].

Thus, the use of mRNA as a vaccine or in treatments seems to be highly effective. This promising mRNA immunization was approved to the first human treatment trial protocol, in which intradermal injections of CV9103 stable mRNA was used against metastatic prostate cancer as well as another strategy using mRNA from tumor as an immune activator [42]. Pascolo and others showed the feasibility and safety of this protocol in humans [43]. These two examples demonstrate that the use of mRNA in gene therapy seems to be one of the easiest, most versatile and theoretically safest technologies in this field. Our results open a perspective to use of this strategy in other infectious disease models with a cost effective when compared with other systems.

## Conclusion

The mRNA based vaccination protocol described here, showed that one immunization with of 10 μg of Hsp65 mRNA administered by the intranasal route could lead suppression of experimental tuberculosis in mice. Since our approach use low dose of a non toxical molecule, delivered by a needle free immunization and is able to establish protection against tuberculosis, it opens a new perspective in gene therapy and can be used in other diseases since is safety, fast and easy to made.

## Methods

### Plasmids

Plasmid pcDNA3A-Hsp65 contained T7 RNA Polymerase promoter and cDNA encoding the gene for *M. Leprae *Hsp65 that has been previously described [20]. Plasmid DNA was purified as described in Endo-Free QIAGEN plasmid purification kit (QIAGEN AG, Basel, Switzerland). Spectrophotometry analysis revealed 260/280 nm ratios of 1.80 or more. The purity of the DNA was confirmed in a 1% agarose gel. Plasmid concentration was determined by spectrophotometry at the wave lengths 260 and 280 nm using Nanodrop ND-1000 (NanoDrop Technologies, Wilmington, USA).

### mRNA preparation

Plasmids containing the full length cDNA of Hsp65 gene (pcDNA3A-Hsp65) were linearized with APA I enzyme and purified at a final concentration of 1 μg/μL by standard protocols. Plasmid pTRI-Xef, used as a control template containing 1.85-kbp *elongation factor 1-α *gene from *Xenopus laevis *(EF-1α), was already linearized by Ambion (Austin, Texas, USA). RNA replicons were *in vitro *transcribed using linearized replicon plasmids and mMessage mMachine Ultra Kit (Ambion), according to the manufacturer's recommendations. After the RNA synthesis was complete, the *in vitro *transcription reactions were treated with RNase-free DNase (Ambion) at 37°C for 15 min to degrade the DNA templates. The next step was the synthesis of poly-A tail, and then the RNA was purified by acidic phenol/chloroform extraction followed by mRNA isopropanol precipitation. mRNAs were quantified by absorbance at 260 and 280 nm using Nanodrop (Thermo), and the proportion of full-length transcripts was checked by formaldehyde denaturing agarose gel electrophoresis. All formulations were tested for endotoxin levels with QCL-1000 Limulus amebocyte lysate. The levels found were under 0.01 EU/mL.

### mRNA structure analysis

Plasmid pcDNA3A-Hsp65 template (data not shown) was sequenced to assure that the sequences required to the perfect translation process were present. Since the plasmid had all these sequences, *in silico *mRNA structure analysis was performed using mFOLD default parameters.

### Cell assay

Transfections was made in HEK 293T cells with mRNA-Hsp65 were made using Transmessenger Transfection Reagent (Qiagen), according to the manufacturer's instructions. Briefly, 10 μg of mRNA-Hsp65 produced *in vitro *was mixed with Transmessenger Reagent for 1 hour and then applied to a 70% confluent culture of HEK 293T cells maintained in Dulbecco's modified Eagle's medium (DMEM) (Gibco-BRL, Gaithersburg, USA) without serum. After 30 minutes, 2, 4, and 8 hours, total RNA was extracted using Trizol (Invitrogen, Carlsbad, USA), according to the manufacturer's recommendation.

### RT-PCR

Total cellular RNA (10 μg/mL) was reverse transcribed using oligo(dT) primers and Superscript reverse transcriptase (Invitrogen), according to the manufacturer's recommendations. The contaminating plasmid DNA was removed by treatment with DNAse I, amplification-grade (Invitrogen). The sequences of PCR primers for the amplification of Hsp cDNA were 5'-ACC AAC GAT GGC GTG TCC AT-3' (sense) and 5'-TAG AAG GCA CAG TCG AGG-3' (antisense), resulting in a 400-bp PCR product. Primers for β-actin were used as a control for the quantity of RNA used. β-actin specific primers amplified a 450-bp product and were composed of the following sequences: 5'-GTG GGC CGC TCT AGG CAC CAA-3' (sense) and 5'-CTC TTT GAT GTC ACG CAC GAT TTC-3' (antisense). All primers were purchased from Invitrogen. The cDNA (2 μg) was submitted to an initial denaturation step (95°C, 5 minutes), followed by 35 cycles of denaturation (94°C, 30 seconds), annealing (60°C, 45 seconds), and extension (72°C, 1.5 minute), and a final extension step (72°C, 3 min). PCR amplification products were analyzed by agarose gel 1% electrophoresis and stained with ethidium bromide. In order to avoid cross-contamination, all procedures including the PCR, were performed in separate laminar flow hoods.

### Western Blot

Total protein content from transfected HEK 293T cells was extracted 30 minutes, 2, 4, and 8 hours after the mRNA transfection using RIPA buffer. The Hsp65 polyclonal antibody and the Hsp65 recombinant protein used in this blot were kindly provided by Dr. Célio Lopes Silva. The total protein extract was separated by SDS-PAGE and transferred to nitrocellulose membranes. Membranes were incubated with blocker solution [(3% BSA (w/v) in PBS containing 0.05% Tween-20 (v/v) (PBS-T)] for 2 hours at 37°C, followed by a incubation with mouse polyclonal anti-Hsp65 IgG for 18 hours at 4°C. After washing with PBS-T, the membranes were incubated with goat anti-mouse IgG peroxidase conjugate (Invitrogen). Reactions were revealed by DAB Substrate Kit for peroxidase (Vector, London, UK).

### mRNA uptake experiments

#### mRNA labeling

mRNA encoding full-length Hsp65 gene was labeled with Alexa Fluor 488 by Universal Linkage System (ULS(tm)) using the ULYSIS nucleic acid labeling kit (Molecular Probe Inc, Eugene, USA) with some modifications. Briefly, mRNA (10 μg) was incubated in labeling buffer (25 μL) and denatured at 95°C for 5 min and cooled on ice. The ULS labeling reagent stock solution (2 μL) was added to the tube and the reaction incubated at 80°C for 15 min. The labeled mRNA was purified by ethanol precipitation, followed by suspension in Ringer Lactate solution (RL).

#### Mice and labeled mRNA immunization

BALB/c mice, 6 to 8 weeks old, were obtained from the Animal Facilities of the Medical School of Ribeirão Preto, University of São Paulo, and were maintained under standard laboratory conditions. The Alexa 488 labeled mRNA (10 μg per mouse in 100 μL volume) formulated in RL was given by intranasal route with 50 μL in each nostril. Control mice were immunized with the same dose of unlabeled mRNA. All experiments were approved and conducted in accordance with the guidelines of the Animal Care Committee of the University.

#### Preparation of lung cells

Lungs were washed with sterile PBS and each was placed in a Petri dish containing incomplete RPMI-1640 medium (Sigma). Lungs were fragmented and transferred to a conical tube containing digestion solution, prepared with Liberase Blendzyme 2 (Roche, Indianapolis, IN) diluted (0·5 μg/ml) in incomplete RPMI-1640. Samples were incubated at 37° under agitation for 30 min After incubation, the cells were dispersed by using a 10-ml syringe and pelleted by centrifugation for 10 min at 400 ***g***. Cells were then washed with complete RPMI-1640, passed through a Nytex screen (Sigma) and resuspended in complete RPMI-1640. Total cell counts were determined in a Neubauer chamber.

#### FACS analysis

Single-cell suspensions (1 × 10^6^) isolated from lungs of immunized mice obtained 30 minutes, 2, 4 and 8 hours after the injection of labeled mRNA were suspended in RPMI 1640 medium (Life Technologies, Grand Island, NY). After lysis of red blood cells (RBCs) with ACK buffer, cells were pre-incubated with anti-CD16/32 (FcBlock - 2.4G2) monoclonal antibodies (mAb) to block Fc*γ*R, and then incubated with the mAb anti-CD19^+ ^(PE), anti-CD11b^+ ^(PerCP) and anti-CD11c^+ ^(APC) for 30 min at 4°C (all antibodies were purchased from Becton Dickinson, San Diego, USA). Mice immunized with unlabeled mRNA were used as a control. Analytical flow cytometry was carried out using a FACSCANTO II (Becton Dickinson, San Jose, USA) and the data were processed using the FACSDIVA software (Becton Dickinson). A biparametric gate was drawn around the mononuclear populations in the forward (FSC) and side (SSC) scatter dot plot. The gated populations were then selected according to CD19^+^, CD11b^+^, or CD11c^+ ^staining. Alexa488 positive population was considered after background analysis from appropriate isotype controls. All antibodies were purchased from BD (BD PharMingen, San Diego, USA). The gating strategy was shown on Additional file 5.

### Mice vaccination and challenge with MTB

#### Animals

Female 6-week-old BALB/c mice were obtained from the Animal Facilities of the Medical School of Ribeirão Preto, University of São Paulo. All experiments were approved and conducted in accordance with the guidelines of the Animal Care Committee of the University. Infected animals were kept in biohazard facility of the Level 3 Biosafety Laboratory and housed in cages within a laminar flow safety enclosure under standard conditions.

#### Immunization procedures

Immunization was performed by the following treatments, using five animals per group. For BCG immunization, one dose of Moreau strain was given by subcutaneous injection of 10^5 ^live bacteria in 100 μL of saline. For mRNA vaccination the mRNA-Hsp65 (10 or 5 μg per mouse in 100 μL volume) was formulated in RL and given by intranasal route with 50 μL drop in each nostril. EF-1α mRNA was given by the same process and formulations. For cytokine evaluation mice were immunized with one dose of mRNA-Hsp65 (10 μg per mouse in 100 μL volume) was formulated in RL and given by intranasal route with 50 μL drop in each nostril, as control we immunize mice only with Ringer solution.

#### Experimental infection with M. tuberculosis

The H37Rv strain of M. tuberculosis (American Type Culture Collection, Rockville, MD) was grown in 7H9 Middlebrook broth (Difco Laboratories, Detroit, USA) for seven days. The culture was harvested through centrifugation and the cell pellet was resuspended in sterile phosphate buffered saline (PBS) and vigorously agitated. The homogeneous suspension was filtered through 2 μm filters (Millipore, Bedford, MA). Viability of the *M. tuberculosis *suspension was pre-tested with fluorescent diacetate (Sigma, Saint Louis, MO) and ethidium bromide at least 80% viable. Thirty days after mRNA immunization all mice groups were challenged with *M. tuberculosis*. Intranasal challenge was made by introducing 1×10^5 ^viable CFU of *M. tuberculosis *H37Rv in 100 μL of PBS by 50 μL drop in each nostril. Control mice received PBS. Mice from all groups were euthanized on day 30 after infection and their lungs were aseptically removed. Lungs from each mouse were used for histopathology analyses and quantification of the bacterial loads.

#### Determination of *M. tuberculosis *CFU in lungs

The number of live bacteria was determined by extracting the right lobes of the lung, washed with sterile PBS, followed by plating 10-fold serial dilutions of homogenized tissue on Middlebrook 7H11 agar media (Difco) [supplemented with 0.2% (v/v) glycerol and 10% (v/v) bovine fetal serum], and counting colonies after 28 days at 37°C. The colony-forming units (CFU) are expressed as log10 of CFU/g lung.

#### Histology

30 days post-infection, the left lobe of each mouse lung was removed and fixed in 10% formalin. Paraffin blocks were prepared and then sectioned for light microscopy. Sections (5 μm each) were stained with hematoxylin & eosin (HE). Slides were evaluated using a Leitz Model Aristoplan microscope (Germany) connected to a Leica Model DFC280 color camera (Heerbrugg, Germany) linked to a PC computer.

#### Evaluation of cytokine production

Two weeks after mRNA-Hsp65 immunization the animals were sacrificed and splenic cells were collected and adjusted to 5 × 10^6 ^cells/ml in RPMI 1640 medium, supplemented with 5% FCS, 20 mM glutamine and 40 IU/l of gentamicin. The cells were cultured in 48-well flat-bottomed culture plates (Nunc, Life Tech. Inc., Maryland, MA, US) in the presence of 20 μg/ml of Concanavalin A (ConA) or Hsp65 recombinant protein. Cytokine levels in culture supernatants were evaluated 48 hours later by ELISA. Cytokines were measured following manufacturer instructions (PharMingen). Purified monoclonal antibodies anti-IFN-γ (R4-6A2) and anti-TNF-alpha (G281-2626) were used at 1 μg/ml as capture antibodies and the following biotinylated antibodies were used for detection: anti-IFN-γ (XMG1.2) and anti-TNF-alpha (MP6-XT3) at 0,5 μg/ml.

#### In vitro TLR 7 assay

Cell Culture, plasmids and transfection. HEK293T cells were grown in DMEM with 10% fetal bovine serum, supplemented with penicillin and streptomycin. All transfections were performed in 24-well plates. One day before transfection, cells were plated at 3 × 10^5 ^cells/well. Cells were transfected with pcDNA3.1 (1 μg/well) as empty vector or the TLRs plasmids (1 μg/well), All TLR vectors was kindly provided by Dr. Aristóbolo Mendes da Silva. The next day cells were left untreated or treated with hsp65 mRNA (2, 5 or 10 μg/ml) or agonists specific for each TLR for 7 h before harvest. The following TLRs agonists were used: poly-IC 100 μg/ml (TLR3), LPS 1 μg/ml (O55:B55) (TLR4) and R-848 1 μg/ml (TLR7 and 8). Nitrite, the end product of NO metabolism, was measured from 50 μl of cell culture supernatants by using Griess reagent, as described elsewhere [44]

#### Statistical analysis

All values are expressed as mean±SEM. Data were investigated by analysis of variance (anova) using graphpad instat software. When the values indicated the presence of a significant difference, a Tukey-Kramer multiple comparisons test was used. Values of *P *< 0·05 were considered significant.

## Authors' contributions

JCCL participated in the design of the study and performed all experiments, APFT and CDR participated in vaccination assays, LP, ICF and AFG carried out DNA sequencing. RL and TM carried out the FACS analyses. JCCL, AMS, LP and TM carried out cytokine evaluation and in vitro TLR7 experiments. RAR carried out RNA structure analysis. AAMCC, AMS and CLS conceived of the study, and participated in its design and coordination and helped to draft the manuscript. All authors read and approved the final manuscript.

## Author Disclosure Statement

No competing financial interests exist.

## Supplementary Material

Additional file 1 **mRNA-Hsp65 structure**. The sequence of Hsp65 ORF plus their 3' UTR region was modeled in Mfold software. This model shows that this mRNA do not have any structural obstacle that can inhibit the translation process. Click here for file

Additional file 2 **Hsp65 expression after mRNA uptake**. After contact mRNA-Hsp65 for different periods of time, the total cell lysate was subjected to polyacrylamide gel electrophoresis (12.5%) and the bands transferred to nitrocellulose membrane and incubated with anti-Hsp65 for 2 hours. The reaction was revealed with secondary antibody anti-mouse IgG in the presence of DAB. The blot was scanned and densitometry analysis was performed using NIH Image J Software. The different time points were shown in the graph. Click here for file

Additional file 3 **Assessment of the amount of non professional antigen-presenting cells (APCs) capable of capturing mRNA-Hsp65 labeled with Alexa488**. Five BALB/c mice per group were immunized by intranasal route with one dose of 10 μg of Alexa488 labeled mRNA-Hsp65. The control group received Ringer's solution. Lung cells were obtained and prepared for flow citometry analysis. The strategy to non APCs was: First of all we make a gating profile that select only the non CD11C, CD11B and CD19 cells, from these cells we make a histogram that measures the alexa 488 positive cells. It is possible to see that non APCs do not capture Alexa488 labeled mRNA-Hsp65. Click here for file

Additional file 4 **Gating strategy**. The cytometry procedure was made to exclude the auto fluorescence and include only Alexa 488 cells. (A). Dot plots for control isotypes on (CD11c^+^, CD11b^+ ^and CD19^+^) and histogram examples gating on Alexa 488 (B). Dot plots for positive cells on (CD11c^+^, CD11b^+ ^and CD19^+^) and histogram for positive cells on Alexa 488. Click here for file

Additional file 5 **Immunostimulatory activity of mRNA-Hsp65**. Five BALB/c mice per group mice were immunized with one dose of mRNA-Hsp65 (10 μg per mouse in 100 μL volume) as control we immunize mice only with Ringer solution. Two weeks later the immunization splenic cells are isolated and stimulated with 20 μg/ml A Concanavalin, 48 hours after stimulation the production of (A) IFN-gamma and (B) TNF-alpha by were determined by ELISA. Click here for file
