# Supplementary figures and images for: Intranasal vaccination with messenger RNA as a new approach in gene therapy: Use against tuberculosis
Source: BMC Biotechnol. 2010 Oct 20;10:77. doi: 10.1186/1472-6750-10-77 (PMC2972232; doi:10.1186/1472-6750-10-77)

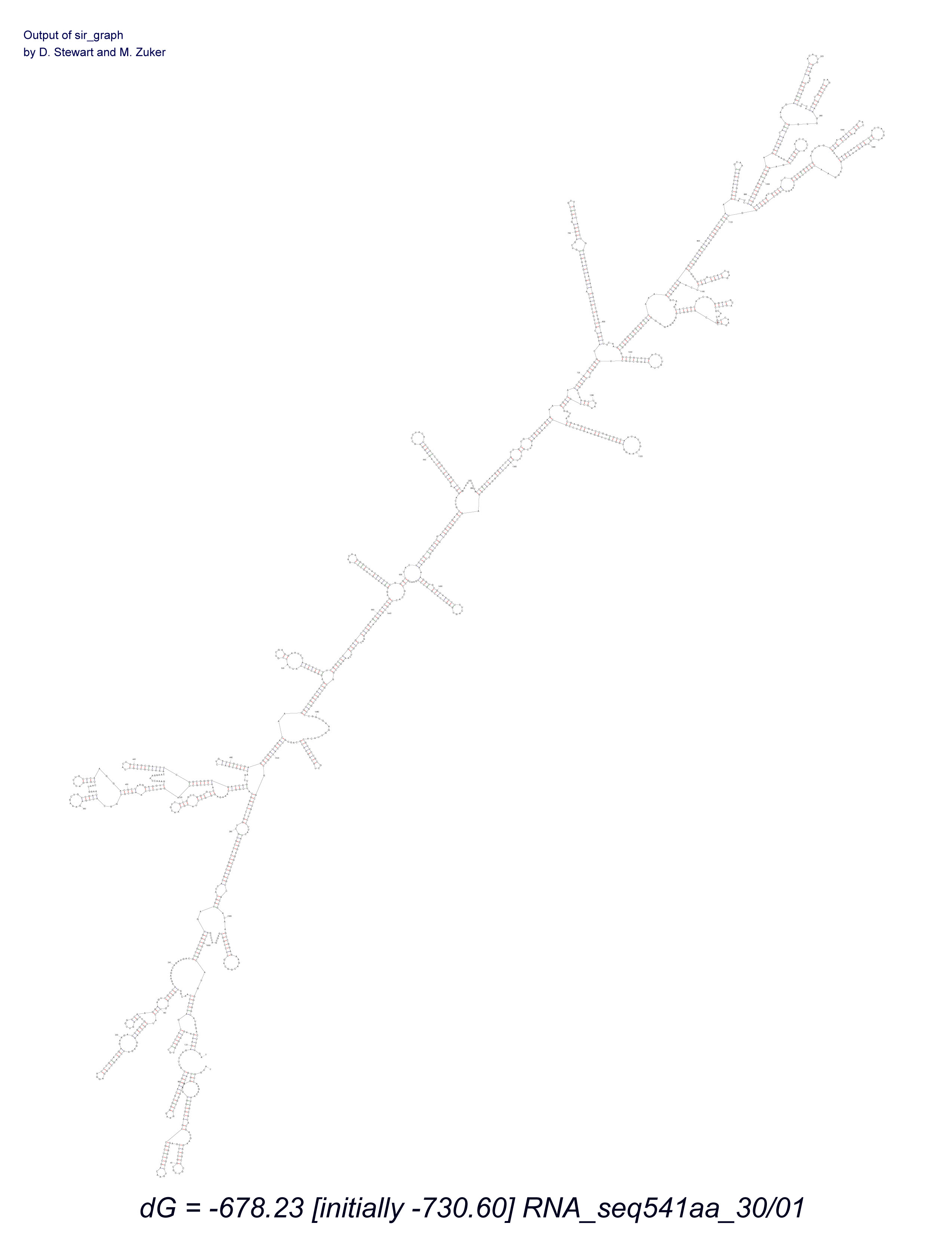

Supplement: Additional file 1 — mRNA-Hsp65 structure. The sequence of Hsp65 ORF plus their 3' UTR region was modeled in Mfold software. This model shows that this mRNA do not have any structural obstacle that can inhibit the translation process. [file 1472-6750-10-77-S1.JPEG]

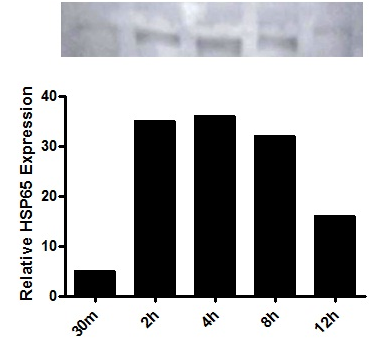

Supplement: Additional file 2 — Hsp65 expression after mRNA uptake. After contact mRNA-Hsp65 for different periods of time, the total cell lysate was subjected to polyacrylamide gel electrophoresis (12.5%) and the bands transferred to nitrocellulose membrane and incubated with anti-Hsp65 for 2 hours. The reaction was revealed with secondary antibody anti-mouse IgG in the presence of DAB. The blot was scanned and densitometry analysis was performed using NIH Image J Software. The different time points were shown in the graph. [file 1472-6750-10-77-S2.TIFF]

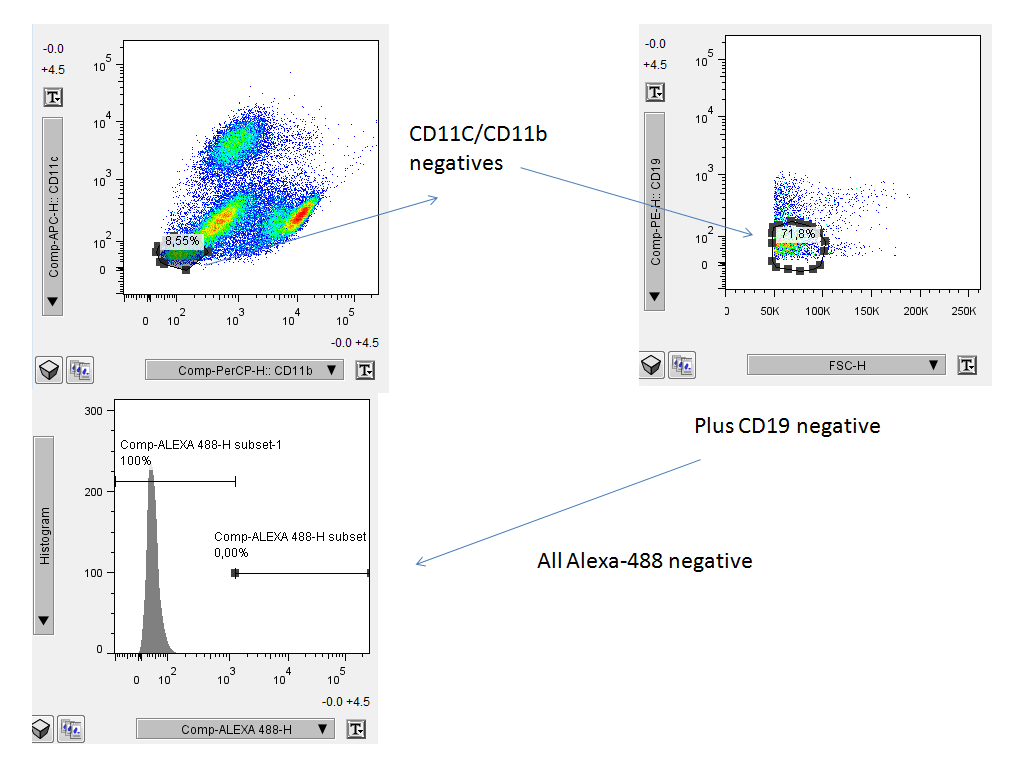

Supplement: Additional file 3 — Assessment of the amount of non professional antigen-presenting cells (APCs) capable of capturing mRNA-Hsp65 labeled with Alexa488. Five BALB/c mice per group were immunized by intranasal route with one dose of 10 μg of Alexa488 labeled mRNA-Hsp65. The control group received Ringer's solution. Lung cells were obtained and prepared for flow citometry analysis. The strategy to non APCs was: First of all we make a gating profile that select only the non CD11C, CD11B and CD19 cells, from these cells we make a histogram that measures the alexa 488 positive cells. It is possible to see that non APCs do not capture Alexa488 labeled mRNA-Hsp65. [file 1472-6750-10-77-S3.TIFF]

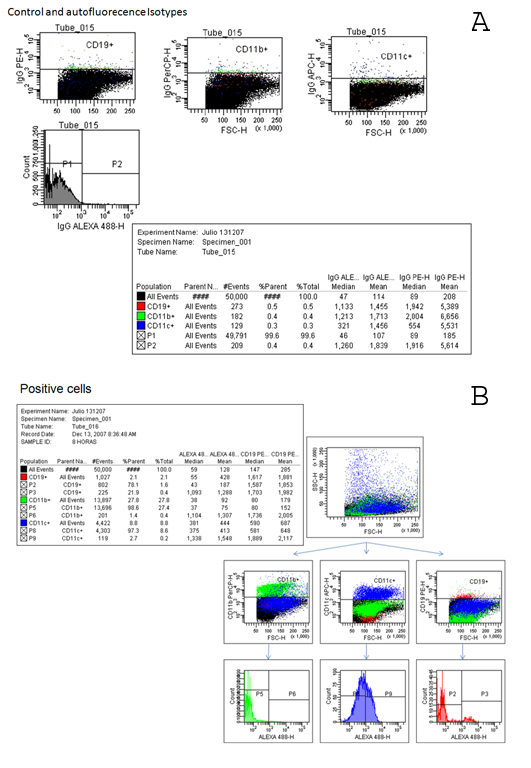

Supplement: Additional file 4 — Gating strategy. The cytometry procedure was made to exclude the auto fluorescence and include only Alexa 488 cells. (A). Dot plots for control isotypes on (CD11c+, CD11b+ and CD19+) and histogram examples gating on Alexa 488 (B). Dot plots for positive cells on (CD11c+, CD11b+ and CD19+) and histogram for positive cells on Alexa 488. [file 1472-6750-10-77-S4.TIFF]

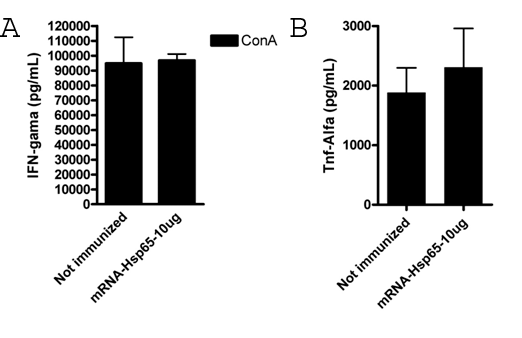

Supplement: Additional file 5 — Immunostimulatory activity of mRNA-Hsp65. Five BALB/c mice per group mice were immunized with one dose of mRNA-Hsp65 (10 μg per mouse in 100 μL volume) as control we immunize mice only with Ringer solution. Two weeks later the immunization splenic cells are isolated and stimulated with 20 μg/ml A Concanavalin, 48 hours after stimulation the production of (A) IFN-gamma and (B) TNF-alpha by were determined by ELISA. [file 1472-6750-10-77-S5.TIFF]
